# Supplementary material for: Chikungunya virus infection in the southernmost state of Brazil was characterised by self-limited transmission (2017-2019) and a larger 2021 outbreak
Source: Mem Inst Oswaldo Cruz. 2023 Jul 31;118:e220259. doi: 10.1590/0074-02760220259 (PMC10392894; doi:10.1590/0074-02760220259)
Supplement: Supplementary file 2 [file 1678-8060-mioc-118-e220259-s2.pdf]

TABLE II

Methods used in Chikungunya virus (CHIKV) diagnosis by real-time quantitative polymerase chain reaction (RT-qPCR) and antibody tests from 2014 to 2022 in Rio Grande do Sul, Brazil

RT-qPCR	Serology	
Samples collected up to eight days from onset symptoms	ELISA IgM: samples collected between 9 and 30 days of onset of symptoms	ELISA IgG: samples collected after 30 days of onset of symptoms
2014-2020<br>In house real time RT-qPCR (LANCIOTTI ET AL. EMERG INFECT DIS. 2007;7:764-767)<br>CHIKV NSP1 874F (5′-AAAGGGCAAACTCAGCTTCAC-3′)<br>CHIKV NSP1 961R (5′-GCCTGGGCTCATCGTTATTC-3′)<br>CHIKV NSP1 899 probe (5′-6FAM/CGCTGTGATACAGTGGTTTCGTGTG/MGBNFQ-3′)<br>CHIKV NSP4 6856F (5′-TCACTCCCTGTTGGACTTGATAGA-3′)<br>CHIKV NSP4 6981R (5′-TTGACGAACAGAGTTAGGAACATACC-3′)<br>CHIKV NSP4 6919 probe (5′-6FAM/AGGTACGCGCTTCAAGTTCGGCG/MGBNFQ-3′)<br>PCR conditions: 30 minutes 50°C, 2 minutes 95°C, and 45 cycles of 15 seconds 95°C and 1 minute 60°C.	2014 - 2019<br>ELISA IgM<br>Anti-CHIKV IgM ELISA<br>(Euroimmun, Germany)	2014 - 2019<br>ELISA IgG<br>Anti-CHIKV IgG ELISA (Euroimmun, Germany)
	2018-2019<br>MAC-ELISA<br>Anti-CHIKV MAC-ELISA<br>(Euroimmun, Germany)	2020<br>ELISA IgG<br>XGEN CHIKUNGUNYA IgG (Mobius Life Science, Paraná, Brazil)
2021-currently<br>Kit molecular ZDC Bio-Manguinhos (Rio de Janeiro, Brazil)<br>PCR conditions: 30 minutes 50°C, 2 minutes 95°C, and 45 cycles of 15 seconds 95°C and 1 minute 60°C.<br><br>Kit Biomol ZDC I-Zika/Dengue/Chikungunya/IBMP (Paraná, Brazil)<br>PCR conditions: 30 minutes 51°C, 15 minutes 95°C, and 40 cycles of 15 seconds 95°C and 1 minute 60°C.	2020-April/2021<br>Anti-CHIKV IgM ELISA<br>Dia.Pro Diagnostics Bioprobes Srl<br>(Milano, Italy)	2021<br>ELISA IgG<br>Anti-CHIKV IgG ELISA (Euroimmun, Germany)
	May 2021-currently<br>Anti-CHIKV IgM ELISA<br>Anti-CHIKV IgM ELISA<br>(Vircell,Spain)	

CC BY

TABLE III

Dataset II used in the Bayesian phylogenetic reconstruction

ID	Location	Location (Brazilian states)	Collection date
ON009842	Brazil	PE	1905-07-07
MG000876	Haiti	Haiti	2016-05-17
MG000875	Haiti	Haiti	2016-06-27
KY704955	Brazil	PB	2016-06-17
KY704954	Brazil	PB	2016-06-20
KU940225	Brazil	BA	2015-06-20
KP164570	Brazil	BA	2014-09-03
KP164568	Brazil	BA	2014-08-26
KP164569	Brazil	BA	2014-08-28
MT526900	Brazil	PA	2017-01-24
KU940226	Brazil	BA	2015-08-01
MH000706	Brazil	PE	2016-02-18
MH000700	Brazil	PE	2016-02-04
KX228391	Brazil	PE	2016-03-03
MH000705	Brazil	PE	2016-02-25
MH000704	Brazil	PE	2016-02-19
MH000703	Brazil	PE	2016-02-15
MH000702	Brazil	PE	2016-02-15
MH823668	Brazil	MT	2017-01-05
MH823667	Brazil	MT	2017-03-01
MH823663	Brazil	MT	2017-01-05
MH823665	Brazil	MT	2017-03-16
MH823666	Brazil	MT	2017-03-01
MH823664	Brazil	MT	2017-03-16
MT526904	Brazil	PA	2017-01-26
MT526901	Brazil	PA	2017-01-25
MT526903	Brazil	PA	2017-01-26
MT526902	Brazil	PA	2017-01-25
MK518395	Brazil	MA	2017-05-16
MK993757	Brazil	RN	2016-01-13
MK993756	Brazil	RN	2016-01-14
MN783353	Brazil	BA	2016-10-13
MN783352	Brazil	BA	2016-04-01
KY704942	Brazil	AL	2016-04-16
KY124328	Brazil	RJ	2016-03-16
KY055011	Brazil	SE	2016-02-20
KY124329	Brazil	RJ	2016-03-16
KY704953	Brazil	AL	2016-04-09
KY704939	Brazil	AL	2016-04-17
OM128439	Brazil	RJ	2016-04-06
OM128438	Brazil	RJ	2016-04-29
OM128437	Brazil	RJ	2018-06-07
OM128433	Brazil	RJ	2018-06-06
OM128436	Brazil	RJ	2018-06-05
OM128434	Brazil	RJ	2018-06-07
OM128431	Brazil	RJ	2018-06-06
OM128440	Brazil	RJ	2018-03-20
MH000701	Brazil	PE	2016-02-22
MT038403	Paraguay	Paraguay	2018-07-10
MT038402	Paraguay	Paraguay	2018-07-03
MT038401	Paraguay	Paraguay	2018-06-25
MT038399	Paraguay	Paraguay	2018-06-25
MT349960	Brazil	MT	2018-02-07
MT038400	Paraguay	Paraguay	2018-06-29
OL898715	Brazil	SP	2021-05-06
OL898687	Brazil	SP	2021-04-16
OL898691	Brazil	SP	2021-02-21
OL898696	Brazil	SP	2021-01-28
OL898693	Brazil	SP	2021-02-13
OL898683	Brazil	SP	2021-01-31
OL898681	Brazil	SP	2021-02-20
OL898680	Brazil	SP	2021-03-22
OL898671	Brazil	SP	2021-02-16
OL898666	Brazil	SP	2021-02-25
OL898665	Brazil	SP	2021-02-22
OL898700	Brazil	SP	2021-01-28
OL898670	Brazil	SP	2021-02-02
OL898676	Brazil	SP	2021-02-02
OL898705	Brazil	SP	2021-02-13
OL898704	Brazil	SP	2021-01-28
OL898688	Brazil	SP	2021-03-23
OL898668	Brazil	SP	2021-01-06
OL898709	Brazil	SP	2021-05-12
OL898667	Brazil	SP	2021-02-01
OL898675	Brazil	SP	2020-12-26
OL898679	Brazil	SP	2021-04-13
OL898664	Brazil	SP	2021-01-31
OL898699	Brazil	SP	2021-02-09
OL898706	Brazil	SP	2021-02-09
OL898672	Brazil	SP	2021-02-25
OL898714	Brazil	SP	2020-11-20
OL898669	Brazil	SP	2020-12-17
OL898684	Brazil	SP	2021-02-14
OL898690	Brazil	SP	2021-01-16
OL898673	Brazil	SP	2021-03-15
OL898710	Brazil	SP	2021-02-02
OL898712	Brazil	SP	2021-02-18
OL898697	Brazil	SP	2021-02-09
OL898702	Brazil	SP	2021-04-27
OL898674	Brazil	SP	2021-01-23
OL898686	Brazil	SP	2021-03-25
OL898711	Brazil	SP	2021-02-08

ID	Location	Location (Brazilian states)	Collection date
OL898707	Brazil	SP	2021-01-07
OL898685	Brazil	SP	2021-02-22
OL898703	Brazil	SP	2021-04-06
OL898701	Brazil	SP	2021-05-05
OL898682	Brazil	SP	2021-01-07
OL898694	Brazil	SP	2021-05-11
OL898698	Brazil	SP	2021-02-25
OL898713	Brazil	SP	2021-04-29
OL898708	Brazil	SP	2021-05-05
OL898663	Brazil	SP	2020-09-05
OL898692	Brazil	SP	2021-03-09
OL898695	Brazil	SP	2021-04-04
OL898678	Brazil	SP	2021-03-05
OL898677	Brazil	SP	2021-02-13
OL898689	Brazil	SP	2021-01-15
MW260519	Brazil	RN	2019-03-30
MW260518	Brazil	RN	2019-03-30
MW260516	Brazil	RN	2019-03-28
MW260513	Brazil	RN	2019-03-23
MW260515	Brazil	RN	2019-03-27
MW260512	Brazil	RN	2019-03-23
MW260514	Brazil	RN	2019-03-28
MK244654	Brazil	RJ	2018-04-06
MK244653	Brazil	RJ	2018-04-06
MK244651	Brazil	RJ	2018-04-05
MK244648	Brazil	RJ	2018-04-04
MK244650	Brazil	RJ	2018-04-03
MK244643	Brazil	RJ	2018-03-28
MK244649	Brazil	RJ	2018-04-03
MT933051	Brazil	RJ	2019-05-28
MT933049	Brazil	RJ	2019-07-07
MT933032	Brazil	RJ	2019-02-05
MT933044	Brazil	RJ	2019-06-13
MT933043	Brazil	RJ	2019-06-07
MT933036	Brazil	RJ	2019-04-17
MT933033	Brazil	RJ	2019-03-08
MT933038	Brazil	RJ	2019-04-22
MT933041	Brazil	RJ	2019-05-08
MT933047	Brazil	RJ	2019-07-02
MT933045	Brazil	RJ	2019-06-19
MT933030	Brazil	RJ	2019-01-28
MT933034	Brazil	RJ	2019-03-15
MT933046	Brazil	RJ	2019-07-01
MK121907	Brazil	RR	2017-02-27
MK121906	Brazil	RR	2017-02-27
MK121904	Brazil	RR	2017-03-02
MK121903	Brazil	RR	2017-03-15

ID	Location	Location (Brazilian states)	Collection date
MK121905	Brazil	RR	2017-03-02
MT933050	Brazil	RJ	2019-08-14
MT933035	Brazil	RJ	2019-04-17
MN428527	Brazil	MT	2018-03-06
MN428525	Brazil	MT	2018-03-06
MN428524	Brazil	MT	2018-03-06
MN428517	Brazil	MT	2018-03-17
MN428519	Brazil	MT	2018-03-16
MN428523	Brazil	MT	2018-03-08
MN428516	Brazil	MT	2018-03-23
MN428526	Brazil	MT	2018-03-06
MN428520	Brazil	MT	2018-03-09
MN428513	Brazil	MT	2018-04-26
MN428507	Brazil	MT	2018-07-05
MN428510	Brazil	MT	2018-04-25
MN428518	Brazil	MT	2018-03-08
MN428521	Brazil	MT	2018-03-16
MN428515	Brazil	MT	2018-03-20
MN428506	Brazil	MT	2018-02-01
MN428514	Brazil	MT	2018-03-13
MN428511	Brazil	MT	2018-04-18
MN428504	Brazil	MT	2018-02-23
MN428522	Brazil	MT	2018-03-08
MN428512	Brazil	MT	2018-03-13
MN428508	Brazil	MT	2018-04-27
MN428505	Brazil	MT	2018-05-21
MT933039	Brazil	RJ	2019-05-06
MT933029	Brazil	RJ	2019-01-21
MT877211	Brazil	CE	2017-06-03
MT877209	Brazil	CE	2017-04-16
MT877208	Brazil	CE	2017-03-22
MT877207	Brazil	CE	2017-03-19
MT877210	Brazil	CE	2017-04-14
MT877206	Brazil	CE	2017-05-30
MT933037	Brazil	RJ	2019-04-18
MW260517	Brazil	RN	2019-03-29
MK121908	Brazil	RR	2017-03-05
MT933042	Brazil	RJ	2019-05-11
MK156063	Brazil	BA	2018-09-03
MK156062	Brazil	BA	2018-08-20
MK156061	Brazil	BA	2018-08-30
MK244641	Brazil	RJ	2017-03-09
MK244635	Brazil	RJ	2016-02-19
MK244642	Brazil	RJ	2018-02-18
1328	Brazil	RS	2017-05-03
MK244640	Brazil	RJ	2017-03-07
MK244634	Brazil	RJ	2016-04-27

ID	Location	Location (Brazilian states)	Collection date
MK244632	Brazil	RJ	2016-04-05
MK156058	Brazil	BA	2018-07-30
MK156055	Brazil	BA	2018-08-20
MK156056	Brazil	BA	2018-08-19
MK156054	Brazil	BA	2018-08-24
MK156059	Brazil	BA	2018-07-27
158	Brazil	RS	2019-02-02
2849	Brazil	RS	2019-06-19
1982	Brazil	RS	2019-05-18
MK244636	Brazil	RJ	2016-05-02
MK244633	Brazil	RJ	2016-05-06
MK244638	Brazil	RJ	2016-04-05
MK244637	Brazil	RJ	2016-05-10
1124	Brazil	RS	2021-03-17
1504	Brazil	RS	2021-03-24
5171	Brazil	RS	2021-04-23
4079	Brazil	RS	2021-04-15
1502	Brazil	RS	2021-03-24
1499	Brazil	RS	2021-03-24
1892	Brazil	RS	2021-03-30
5180	Brazil	RS	2021-04-26
4081	Brazil	RS	2021-04-15
1509	Brazil	RS	2021-03-24
5166	Brazil	RS	2021-04-23
6165	Brazil	RS	2021-05-07
4080	Brazil	RS	2021-04-16
7302	Brazil	RS	2021-05-13
6701	Brazil	RS	2021-05-12
5183	Brazil	RS	2021-04-27
2405	Brazil	RS	2021-03-31
MT933048	Brazil	RJ	2019-07-31
OM128432	Brazil	RJ	2018-03-26
KY704952	Brazil	AL	2016-04-07
KY704948	Brazil	AL	2016-04-19
KY704933	Brazil	AL	2016-03-30
MK121895	Brazil	AM	2017-03-20
MK121893	Brazil	AM	2015-07-15
MK121891	Brazil	AM	2015-07-15
MK121902	Brazil	RR	2017-03-17
MK121900	Brazil	RR	2017-03-17
MK121899	Brazil	RR	2017-03-17
MK121898	Brazil	RR	2017-02-22
MK121896	Brazil	RR	2017-03-03
MK156064	Brazil	BA	2018-09-03
MK156053	Brazil	BA	2018-08-24
1115	Brazil	RS	2021-03-17
MK244656	Brazil	RJ	2018-04-06
MK244652	Brazil	RJ	2018-04-05
MK244655	Brazil	RJ	2018-04-06
MK244644	Brazil	RJ	2018-03-26
MK244647	Brazil	RJ	2018-03-27
MK244646	Brazil	RJ	2018-03-28
MK244645	Brazil	RJ	2018-03-28
MK156060	Brazil	BA	2018-08-02
MT933031	Brazil	RJ	2019-02-18
MT933040	Brazil	RJ	2019-05-06
MN428509	Brazil	MT	2018-03-13
KY704946	Brazil	AL	2016-04-17
KY704935	Brazil	AL	2016-04-01
KY704941	Brazil	AL	2016-04-14
KY704950	Brazil	AL	2016-04-07
KY704938	Brazil	AL	2016-04-19
KY704944	Brazil	AL	2016-04-19
KY704934	Brazil	AL	2016-03-30
336	Brazil	RS	2019-03-06
OM128435	Brazil	RJ	2018-03-19
KY704940	Brazil	AL	2016-04-14
MK244639	Brazil	RJ	2016-04-19
KY704936	Brazil	AL	2016-04-01

TABLE IV
Chikungunya fever (CHIKF) reported symptoms distribution per different categories

Clinical manifestations	Cases	Category
Petechiae	2	Notified
Arthritis	7	Notified
Conjunctivitis	9	Notified
Leukopenia	11	Notified
Retro orbital pain	37	Notified
Vomiting	47	Notified
Back pain	96	Notified
Nausea	104	Notified
Myalgia	118	Notified
Rash	133	Notified
Headache	177	Notified
Fever	323	Notified
Intense arthralgia	387	Notified
Leukopenia	0	Confirmed
Petechiae	1	Confirmed
Conjunctivitis	3	Confirmed
Arthritis	4	Confirmed
Vomiting	20	Confirmed
Retro orbital pain	20	Confirmed
Back pain	39	Confirmed
Nausea	48	Confirmed
Myalgia	53	Confirmed
Rash	81	Confirmed
Headache	89	Confirmed
Fever	189	Confirmed
Intense arthralgia	225	Confirmed
Leukopenia	0	Discarded
Petechiae	1	Discarded
Arthritis	3	Discarded
Conjunctivitis	6	Discarded
Retro orbital pain	17	Discarded
Vomiting	27	Discarded
Rash	52	Discarded
Nausea	55	Discarded
Back pain	57	Discarded
Myalgia	64	Discarded
Headache	87	Discarded
Fever	133	Discarded
Intense arthralgia	161	Discarded

TABLE V

tMRCA Bayesian reconstruction of specific clades depicted in Fig. 3

Clades	Height	Height_95	Month height	Month height_95
Clade 1	5,1179	4,9269 - 5,3597	Apr 16	Jan 2016 - Jun 2016
Clade 2	5,0048	4,6672 - 5,3684	May 16	Jan 2016 - Oct 2016
Clade3	3,4184	3,2221 - 3,6398	Dec 17	Oct 2017 - Fev 2018
Clade4	2,45	1,9942 - 2,9212	Nov 18	Jun 2018 - May 2019
Clade4 RS/SP ancestral	1,5072	0,9716 - 1,9738	Sep 19	May 2019 - May 2020
Clade 4 RS ancestral	0,5549	0,3346 - 0,8478	Oct 20	Jul 2020 - Jan 2021

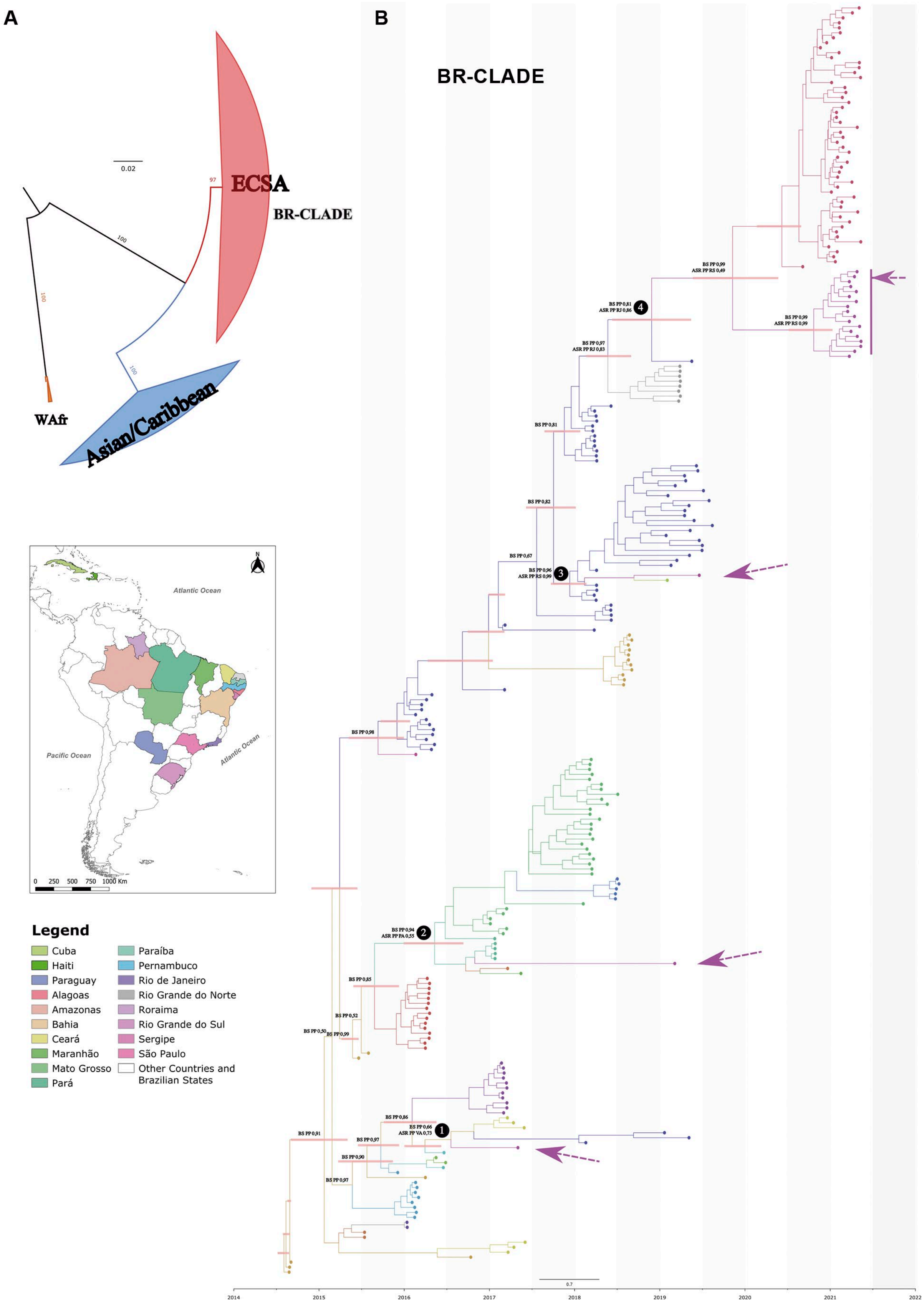

Phylogenetic trees of full Chikungunya genomes depicting the three known Chikungunya genotypes (A) and the East-Central-South-African (ECSA) BR-CLADE time tree reconstructed under a Bayesian framework (B). BSPP: Bayesian support posterior probability; ASRPP: ancestral state reconstruction posterior probability.
